# Variation in initial biopsy technique for primary melanoma diagnosis: A population-based cohort study in New South Wales, Australia

**DOI:** 10.1016/j.jdin.2024.08.024

**Published:** 2024-10-18

**Authors:** Kathy Dempsey, Genevieve Ho, Serigne N. Lo, Janet McKeown, Caroline G. Watts, Anne E. Cust, Pascale Guitera, Richard A. Scolyer, John F. Thompson, Rachael L. Morton, MPOC (Melanoma Patterns of Care) Study Group, Scott Menzies, Christine Madronio, Graham Mann

**Affiliations:** i Sydney Melanoma Diagnostic Centre, Royal Prince Alfred Hospital, Sydney, Australia; b Melanoma Institute Australia, The University of Sydney, Sydney, Australia; c The University of Sydney, Sydney, Australia; h Department of Melanoma and Surgical Oncology, Royal Prince Alfred Hospital, Sydney, Australia; a NHMRC Clinical Trials Centre, Faculty of Medicine and Health, The University of Sydney, Sydney, Australia; b Melanoma Institute Australia, The University of Sydney, Sydney, Australia; c The University of Sydney, Sydney, Australia; d The Daffodil Centre, The University of Sydney and Cancer Council NSW, Sydney, Australia; e Tissue Pathology and Diagnostic Oncology, Royal Prince Alfred Hospital, Sydney, Australia; f NSW Health Pathology, Sydney, Australia; g Charles Perkins Centre, The University of Sydney, Sydney, Australia; h Department of Melanoma and Surgical Oncology, Royal Prince Alfred Hospital, Sydney, Australia

**Keywords:** biopsy, cohort study, health services research, melanoma

## Abstract

**Background:**

Factors associated with nonadherence to guideline-recommended complete excision of suspicious cutaneous lesions are unclear.

**Objective:**

The purpose of this study was to analyze patient, melanoma, and clinician factors associated with initial diagnostic biopsy type and determine whether unwarranted variation from guidelines occurred.

**Methods:**

This population-based, cohort study involved the analysis of data from questionnaires completed by clinicians who managed patients with newly diagnosed, histopathologically confirmed primary invasive cutaneous melanomas reported to the New South Wales Cancer Registry between 2006 and 2007.

**Results:**

Of the 2267 biopsies, complete excision was attempted in 69.1% of cases but histologically incomplete in 14.0%. Multivariable regression analyses showed that complete excision was more likely than incision biopsy in patients <70 years (*P* = .016), shave biopsy in patients <80 years (*P* = .034), shave biopsy in melanomas of Breslow thickness 0.8-1.0 mm or 2.1-4.0 mm (*P* = .039) than either punch (*P* < .001) or shave biopsy (*P* < .003) in melanomas on trunk or limbs, and punch biopsy when treated by a surgeon (*P* < .001). Complete excision was less likely than punch biopsies in women (*P* < .003), with lentigo maligna melanoma or unknown histopathology (*P* = .004); shave biopsy in patients with lentigo maligna melanoma, or other melanoma subtype (*P* = .003); punch, shave, or incision biopsy when treated by a dermatologist (*P* < .001).

**Limitations:**

Generalizability of these findings may be limited to the time of data collection.

**Conclusions:**

Guideline adherence for biopsy type undertaken for clinically suspected melanoma appeared to be suboptimal.

**Capsule Summary**

• Is there unwarranted variation in initial biopsy technique for primary melanoma diagnosis?
• In this population-based cohort study, one-quarter of biopsies performed for clinically suspicious melanomas were partial biopsies that did not adhere to recommended guidelines. Future research should focus on specific enablers to enhance guideline adherence.

## Introduction

Early and accurate diagnosis of melanomas contributes to improved patient outcomes^1^^,^^2^ and reduced costs to health systems.^3^ One factor associated with diagnostic inaccuracy for early-stage invasive melanoma is the type of initial biopsy performed, with increases in partial biopsies leading to a greater risk of diagnostic inaccuracies.^4^

Best-practice guidelines for melanoma diagnosis in Australia were determined by an expert review of the most recently available high-quality evidence.^5^ The 1999 guidelines, in use at the time of data collection, stated: “Shave and punch biopsies of pigmented lesions should be avoided if excision biopsy with primary skin closure can be achieved.”^6^ The current (2018) guidelines are more specific, stating the optimal biopsy approach for a suspicious pigmented cutaneous lesion is complete excision with a 2-mm clinical margin and upper subcutis.^5^ These guidelines also acknowledge partial biopsies (incisional, punch, or shave) may sometimes be appropriate in carefully selected clinical circumstances (large in situ lesions, large facial, or acral lesions, or in which the suspicion of melanoma is low) on the proviso that these partial biopsies be undertaken by experienced clinicians.^5^ We define an excision biopsy as the removal of an elliptical piece of skin to the level of subcutaneous fat and closed with sutures. An incisional biopsy removes a portion of the lesion including a 1 mm area of normal-appearing skin adjacent at one end of the ellipse and extending to the subcutaneous fat.

Unwarranted clinical variation in health outcomes is a major issue for the Australian health care system, which provides services for a geographically dispersed and socioeconomically diverse population. Cancer Council New South Wales (NSW) identified “distance to care” as the most significant barrier to accessing cancer care generally.^7^ Melanoma incidence rates are higher in regional and remote areas than in major cities,^8^ and the problem is compounded by later diagnosis, with a study in rural Victoria reporting older men in regional areas presented with later stage melanoma than their metropolitan counterparts and had poorer survival rates.^9^ Another study, randomly sampling 400 patients from the Victorian Cancer Registry with melanoma diagnosed in the years 2005, 2010, and 2015, documented a sharp increase in the use of shave biopsies to diagnose invasive melanomas, from 9% in 2005 to 15% in 2015. However, this study did not consider potential access to best-practice issues such as patient socio-economic status (SES) and remoteness.^10^

We used data from the population-based Melanoma Patterns of Care (MPOC) study in NSW to explore 2 aims: first, to examine patient, melanoma, and clinician factors associated with the type of initial melanoma diagnostic biopsy, and second, to determine whether unwarranted variation from national biopsy guidelines occurred, particularly in relation to SES, remoteness, and/or access to melanoma specialists.

## Methods

### Study design

This population-based, cohort study involved data analysis from questionnaires completed by general practitioners (GPs) and specialists, indicating their management of melanoma patients residing in NSW. Eligible clinicians were those who managed patients with newly diagnosed, histopathologically confirmed primary invasive cutaneous melanomas reported to the NSW Cancer Registry between 23 October 2006 and 22 October 2007. Pathologists in NSW are required by law to notify the state Cancer Registry of every newly diagnosed cancer. Patients were followed up for 12 months until their surgical management had been completed.

Patients’ demographic information was obtained from the Cancer Registry. Questionnaires were sent directly to the clinicians via the Registry, with a 78% response rate.^11^ An incision was defined in the questionnaire by the following 2 questions: “*Did you do a partial biopsy?* (No; Yes); *If yes, please specify type*; (punch biopsy; shave biopsy; and incision biopsy).” Further information about the original study, which was approved by the Human Research Ethics Committees of the University of Sydney and Cancer Institute NSW, has been published elsewhere.^11^ See Supplementary Appendix A, available via Mendeley at https://data.mendeley.com/datasets/4ghbckk9xp/3 for the questionnaire sent to GPs and Supplementary Appendix B, available via Mendeley at https://data.mendeley.com/datasets/4ghbckk9xp/3 for the questionnaire sent to specialist clinicians.

### Statistical analysis

When available, questionnaire data were prioritized over pathology data because the main outcome of interest was the clinician-reported type of biopsy. When a patient had >1 melanoma, data from the thickest melanoma were analyzed. The variables investigated were age at the time of diagnosis, sex, socio-economic disadvantage of area of residence and of clinic location (socio-economic indexes for areas index of relative socio-economic disadvantage),^12^ remotenesses of patient residence and clinic location (Australian Statistical Geography Standard Remoteness Structure),^13^ clinically melanoma (yes/suspicious, no), melanoma subtype, lesion site, Breslow thickness in millimeters, clinician category, practice setting, and patient referred before biopsy (yes/no). Clinician-reported biopsy type was divided into excision biopsy (including complete excision attempted and complete excision achieved) and punch, shave, or incision.

Continuous variables were summarized with means (SD), medians (range), and interquartile range. Categorical variables were described using frequencies and proportions. All variables were stratified by biopsy type and clinical suspicion of melanoma (yes or suspicious, vs no). Differences between groups were tested using *t* test for continuous variables and chi-square test for categorical variables. Associations between biopsy type (categorized in 6 paired biopsy comparisons: excision vs punch, excision vs shave, excision vs incision, incision vs punch, incision vs shave, and shave vs punch) and the selected variables were evaluated using univariate and multivariable logistic regression analyses. The multivariable model included age, sex, socio-economic and remoteness variables, and any other variable with a *P* value < .20 from the univariate analysis. Variables with a missing proportion >10% were not included in multivariable models. Statistical significance was set to a 2-sided *P* < .05. All analyses were performed using SAS version 9.4 software.^14^

## Results

### Patient demographics

Mean patient age was 63.2 years, with 45% aged between 60 and 79 years, and just over 60% of patients were men. Most patients resided in the least disadvantaged quintile, with the vast majority living in major cities or inner regional areas. GPs comprised over half of the clinicians who performed the biopsies, and slightly more patients were seen in nonspecialist settings, located in the middle or least disadvantaged area quintiles. Fourteen percent of patients living in outer regional or remote areas were diagnosed with melanomas.

### Biopsies

Of 2267 biopsies, 72.9% were excision. Just under one-quarter (24%) of biopsies for clinically suspicious melanomas were partial biopsies including punch, shave, or incision biopsies that were not recommended in the guidelines. Complete excision was attempted in 69.1% of cases but histologically incomplete in 14.0%. (Table I). Sixty-seven percent of clinicians attempted the guideline-recommended complete excision biopsy, with over 56% reporting they achieved a complete excision.

**Table I:** Association between biopsy type and patient, melanoma, and clinician characteristics

Characteristics	Overall	Punch	Shave	Excision	Incision	Missing	*P* value
	(*N* = 2267)	(*N* = 292)	(*N* = 191)	(*N* = 1652)	(*N* = 86)	(*N* = 46)	
Age, y, mean (SD)	63.20 (15.76)	64.30 (14.44)	67.90 (13.81)	62.30 (16.21)	68.60 (13.17)	58.0 (28.4)	<.0001
Age categories (y)							
<40	200 (8.8%)	14 (4.8%)	6 (3.1%)	175 (10.6%)	3 (3.5%)	2 (4.3%)	<.0001
40-49	251 (11.1%)	33 (11.3%)	18 (9.4%)	185 (11.2%)	5 (5.8%)	10 (21.7%)	
50-59	421 (18.6%)	54 (18.5%)	27 (14.1%)	315 (19.1%)	13 (15.1%)	12 (26.1%)	
60-69	507 (22.4%)	78 (26.7%)	40 (20.9%)	363 (22.0%)	17 (19.8%)	9 (19.6%)	
70-79	521 (23.0%)	68 (23.3%)	56 (29.3%)	356 (21.5%)	30 (34.9%)	11 (23.9%)	
80+	367 (16.2%)	45 (15.4%)	44 (23.0%)	258 (15.6%)	18 (20.9%)	2 (4.3%)	
Sex							
Males	1392 (61.4%)	149 (51.0%)	121 (63.4%)	1035 (62.7%)	55 (64.0%)	32 (69.6%)	.0028
Females	875 (38.6%)	143 (49.0%)	70 (36.6%)	617 (37.3%)	31 (36.0%)	14 (30.4%)	
Breslow thickness (mm)							
0.1-0.7	1219 (54.0%)	150 (51.4%)	130 (69.1%)	873 (53.0%)	46 (54.1%)	20 (44.4%)	.0054
0.8-1.0	307 (13.6%)	47 (16.1%)	18 (9.6%)	227 (13.8%)	11 (12.9%)	4 (8.9%)	
1.1-2.0	382 (16.9%)	56 (19.2%)	28 (14.9%)	273 (16.6%)	12 (14.1%)	13 (28.9%)	
2.1-4.0	230 (10.2%)	26 (8.9%)	9 (4.8%)	180 (10.9%)	9 (10.6%)	6 (13.3%)	
>4.0	120 (5.3%)	13 (4.5%)	3 (1.6%)	95 (5.8%)	7 (8.2%)	2 (4.4%)	
Missing	9 (0.40%)	0 (0.0%)	3 (1.6%)	4 (0.2%)	1 (1.2%)	0 (0.0%)	
Melanoma subtype							
SSM	1192 (52.6%)	135 (46.2%)	80 (41.9%)	909 (55.0%)	44 (51.2%)	24 (52.2%)	<.0001
NM	297 (13.1%)	30 (10.3%)	19 (9.9%)	235 (14.2%)	7 (8.1%)	6 (13.0%)	
LMM	238 (10.5%)	47 (16.1%)	39 (20.4%)	138 (8.4%)	13 (15.1%)	1 (2.2%)	
Other	131 (5.8%)	17 (5.8%)	14 (7.3%)	89 (5.4%)	5 (5.8%)	6 (13.0%)	
Not stated	409 (18.0%)	63 (21.6%)	39 (20.4%)	281 (17.0%)	17 (19.8%)	9 (19.6%)	
Lesion site							
Head and neck	414 (18.3%)	88 (30.1%)	49 (25.7%)	250 (15.1%)	21 (24.4%)	6 (13.0%)	<.0001
Anterior trunk	159 (7.0%)	17 (5.8%)	6 (3.1%)	118 (7.1%)	12 (14.0%)	6 (13.0%)	
Posterior trunk	740 (32.6%)	67 (22.9%)	72 (37.7%)	564 (34.1%)	21 (24.4%)	16 (34.8%)	
Upper limb	501 (22.1%)	55 (18.8%)	40 (20.9%)	378 (22.9%)	14 (16.3%)	14 (30.4%)	
Lower limb	430 (19.0%)	64 (21.9%)	24 (12.6%)	321 (19.4%)	17 (19.8%)	4 (8.7%)	
Missing	23 (1.0%)	1 (0.3%)	0 (0.0%)	21 (1.3%)	1 (1.2%)	0 (0.0%)	
Clinician type							
GP	1186 (52.3%)	158 (54.1%)	33 (17.3%)	950 (57.5%)	35 (40.7%)	10 (21.7%)	<.0001
Dermatologist	594 (26.2%)	104 (35.6%)	143 (74.9%)	297 (18.0%)	40 (46.5%)	10 (21.7%)	
Surgeon	325 (14.3%)	19 (6.5%)	8 (4.2%)	272 (16.5%)	5 (5.8%)	21 (45.7%)	
Plastic surgeon	149 (6.6%)	9 (3.1%)	7 (3.7%)	122 (7.4%)	6 (7.0%)	5 (10.9%)	
Other/missing	13 (0.6%)	2 (0.1%)	0 (0.0%)	11 (0.5%)	0 (0.0%)	0 (0.0%)	
Clinically melanoma							
No	378 (16.7%)	73 (25.0%)	42 (22.0%)	237 (14.4%)	15 (17.4%)	11 (27.5%)	<.0001
Yes or suspicious	1812 (79.9%)	210 (71.9%)	147 (77.0%)	1356 (82.1%)	70 (81.4%)	29 (72.5%)	
Missing	77 (3.4%)	9 (3.1%)	2 (1.1%)	59 (3.6%)	1 (1.2%)	0 (0.0%)	
Complete excision attempted							
No	678 (30.0%)	224 (76.7%)	155 (81.2%)	196 (11.9%)	79 (91.9%)	24 (52.2%)	<.0001
Yes	1513 (66.7%)	57 (19.5%)	34 (17.8%)	1394 (84.4%)	7 (8.1%)	21 (45.7%)	
Missing	76 (3.4%)	11 (3.8%)	2 (1.1%)	62 (3.8%)	0 (0.0%)	1 (2.1%)	
Complete excision achieved							
No	208 (9.2%)	11 (3.8%)	12 (6.3%)	177 (10.7%)	5 (5.8%)	3 (6.5%)	<.0001
Yes	1274 (56.2%)	42 (14.4%)	21 (11.0%)	1191 (72.1%)	2 (2.3%)	18 (39.1%)	
Missing	785 (34.6%)	239 (81.9%)	158 (82.7%)	284 (17.2%)	79 (91.9%)	25 (54.3%)	
Partial biopsy∗							
No	615 (27.1%)	29 (9.9%)	22 (12.9%)	538 (32.6%)	0 (0.0%)	26 (56.5%)	<.0001
Yes	471 (20.8%)	212 (72.6%)	148 (87.1%)	22 (1.3%)	86 (100%)	3 (6.5%)	
Missing	1188 (52.1%)	51 (17.4%)	21 (11.0%)	1092 (66.1%)	0 (0.0%)	17 (37.0%)	

Chi-squared test for association (please note missing values excluded from chi-squared test). Percentages may not sum to 100 due to rounding. A *P* value of < .05 is considered significant.

*LMM*, Lentigo maligna melanoma; *NM*, nodular melanoma; *SSM*, superficial spreading melanoma.

∗ Partial biopsy includes punch, shave, or incision biopsy.

Eighty percent of cases were clinically suspicious of melanoma The majority of invasive melanomas were thin (67.6% were ≤ 1.0 mm); occurred on the posterior trunk and were of superficial spreading subtype.

### Associations with type of biopsy

All assessed variables (patient, melanoma, and clinician characteristics) were significantly associated with the type of biopsy (Table I), with the exception of whether the treating clinician referred the patient to another clinician prior to biopsy.

Table II used chi-square analyses to provide descriptive associations between patient, melanoma, clinician, and biopsy characteristics stratified by clinical suspicion of melanoma (yes or suspicious, vs no). Significant differences were found for age (*P* = .030), Breslow thickness (*P* < .001), biopsy type (*P* < .001), complete excision attempted (*P* < .001), partial biopsy performed (*P* = .004), nonmelanocytic lesions (*P* < .001), all clinician categories (*P* = .015), observing lesion before biopsy (*P* < .001), and histopathology (*P* < .001).

**Table II:** Association between patient, melanoma, clinician, and biopsy characteristics stratified by melanoma (yes or suspicious vs no)∗

Characteristics	Overall	No	Yes/suspicious	*P* value
	(*N* = 2267)	(*N* = 378)	(*N* = 1812)	
Age, y, mean (SD)	63.3 (15.8)	64.9 (15.2)	63.0 (15.9)	.030
Age categories (y)				
<40	195/2190 (8.9%)	21/378 (5.6%)	174/1812 (9.6%)	.028
40-49	238/2190 (10.9%)	47/378 (12.4%)	191/1812 (10.5%)	
50-59	405/2190 (18.5%)	66/378 (17.5%)	339/1812 (18.7%)	
60-69	490/2190 (22.4%)	87/378 (23.0%)	403/1812 (22.2%)	
70-79	500/2190 (22.8%)	79/378 (20.9%)	421/1812 (23.2%)	
80+	362/2190 (16.5%)	78/378 (20.6%)	284/1812 (15.7%)	
Sex				
Males	1351/2190 (61.7%)	220/378 (58.2%)	1131/1812 (62.4%)	.125
Females	839/2190 (38.3%)	158/378 (41.8%)	681/1812 (37.6%)	
Breslow thickness (mm)				
0.1-0.7	1179/2181 (54.1%)	125/373 (33.5%)	1054/1808 (58.3%)	<.001
0.8-1.0	299/2181 (13.7%)	56/373 (15.0%)	243/1808 (13.4%)	
1.1-2.0	366/2181 (16.8%)	73/373 (19.6%)	293/1808 (16.2%)	
2.1-4.0	218/2181 (10.0%)	72/373 (19.3%)	146/1808 (8.1%)	
>4.0	119/2181 (5.5%)	47/373 (12.6%)	72/1808 (4.0%)	
Melanoma subtype				
SSM	1152/2190 (52.6%)	165/378 (43.7%)	987/1812 (54.5%)	<.001
NM	287/2190 (13.1%)	89/378 (23.5%)	198/1812 (10.9%)	
LMM	227/2190 (10.4%)	26/378 (6.9%)	201/1812 (11.1%)	
Other	121/2190 (5.5%)	40/378 (10.6%)	81/1812 (4.5%)	
Not stated	403/2190 (18.4%)	58/378 (15.3%)	345/1812 (19.0%)	
Lesion site				
Head and neck	398/2190 (18.2%)	85/378 (22.5%)	313/1812 (17.3%)	.111
Anterior trunk	155/2190 (7.1%)	20/378 (5.3%)	135/1812 (7.5%)	
Posterior trunk	720/2190 (32.9%)	111/378 (29.4%)	609/1812 (33.6%)	
Upper limb	482/2190 (22.0%)	88/378 (23.3%)	394/1812 (21.7%)	
Lower limb	420/2190 (19.2%)	72/378 (19.0%)	348/1812 (19.2%)	
Missing	15/2190 (0.7%)	2/378 (0.5%)	13/1812 (0.7%)	
Nonmelanocytic lesion				
No	214/329 (65.0%)	16/84 (19.0%)	198/245 (80.8%)	<.001
Yes	115/329 (35.0%)	68/84 (81.0%)	47/245 (19.2%)	
Biopsy				
Excision	1593/2177 (73.2%)	237/377 (62.9%)	1356/1800 (75.3%)	<.001
Punch	283/2177 (13.0%)	73/377 (19.4%)	210/1800 (11.7%)	
Shave	189/2177 (8.7%)	42/377 (11.1%)	147/1800 (8.2%)	
Incision	85/2177 (3.9%)	15/377 (4.0%)	70/1800 (3.9%)	
Other and Unknown	27/2177 (1.2%)	10/377 (2.7%)	17/1800 (0.9%)	
Complete excision attempted				
No	622/2122 (29.3%)	142/364 (39.0%)	480/1758 (27.3%)	<.001
Yes	1500/2122 (70.7%)	222/364 (61.0%)	1278/1758 (72.7%)	
Excision completed				
No	205/1469 (14.0%)	38/215 (17.7%)	167/1254 (13.3%)	.088
Yes	1264/1469 (86.0%)	177/215 (82.3%)	1087/1254 (86.7%)	
Partial biopsy∗				
No	560/1027 (54.5%)	91/200 (45.5%)	469/827 (56.7%)	.004
Yes	467/1027 (45.5%)	109/200 (54.5%)	358/827 (43.3%)	

Chi-squared test for association (please note missing values excluded from chi-squared test). Percentages may not sum to 100 due to rounding. A *P* value of <.05 is considered significant.

*LMM*, Lentigo maligna melanoma; *NM*, nodular melanoma; *SSM*, superficial spreading melanoma.

∗ Partial biopsy includes punch, shave, or incision biopsy.

Univariate analyses are shown in Table III. All variables were significant for 1 or more biopsy types, with practice setting and clinician category being significant for all paired biopsy comparisons except incision vs punch. However, to alleviate potential multicollinearity issues in the multivariable analysis between practice setting and clinician type, the variable that showed the highest *P* value was excluded.

**Table III:** Univariate logistic regression of biopsy type for clinical melanoma (yes/suspicious), *n* = 1812

Characteristic	Excision vs punch *(Ref = punch)*	Excision vs shave *(Ref = shave)*	Excision vs incision *(Ref = incision)*	Incision vs punch *(Ref = punch)*	Incision vs shave *(Ref = shave)*	Shave vs punch *(Ref = punch)*
Age (y)	*P = .154*	*P < .001*	*P = .004*	*P = .080*	*P = .984*	*P = .036*
<60	1	1	1	1	1	1
60-69	0.66 (0.45, 0.97)	0.59 (0.36, 0.98)	0.52 (0.25, 1.08)	1.27 (0.57, 2.81)	1.13 (0.48, 2.68)	1.12 (0.62, 2.04)
70-79	0.73 (0.49, 1.08)	0.37 (0.24, 0.59)	0.32 (0.17, 0.62)	2.27 (1.08, 4.76)	1.17 (0.54, 2.54)	1.95 (1.11, 3.43)
80+	0.77 (0.49, 1.19)	0.38 (0.23, 0.62)	0.33 (0.16, 0.69)	2.30 (1.01, 5.19)	1.13 (0.48, 2.65)	2.03 (1.08, 3.81)
Sex	*P < .002*	*P = .665*	*P = .884*	*P = .129*	*P = .893*	*P = .075*
Males	1	1	1	1	1	1
Females	0.63 (0.47, 0.84)	0.93 (0.65, 1.31)	0.96 (0.59, 1.58)	0.65 (0.37, 1.13)	0.96 (0.53, 1.73)	0.68 (0.44, 1.04)
Breslow thickness	*P = .718*	*P = .001*	*P = 1.000*	*P = .894*	*P = .036*	*P = .008*
0.1-0.7 mm	1	1	1	1	1	1
0.8-1.0 mm	0.86 (0.57, 1.30)	2.70 (1.38, 5.25)	0.93 (0.46, 1.90)	0.92 (0.42, 2.04)	2.90 (1.12, 7.50)	0.32 (0.15, 0.67)
1.1-2.0 mm	0.94 (0.63, 1.41)	1.74 (1.04, 2.93)	0.98 (0.50, 1.96)	0.96 (0.44, 2.06)	1.77 (0.77, 4.08)	0.54 (0.29, 1.01)
2.1-4.0 mm	1.16 (0.66, 2.06)	2.68 (1.15, 6.25)	0.79 (0.35, 1.82)	1.46 (0.56, 3.85)	3.38 (1.07, 10.68)	0.43 (0.16, 1.16)
>4.0 mm	1.54 (0.65, 3.65)	4.28 (1.03, 17.74)	0.98 (0.30, 3.28)	1.57 (0.37, 6.57)	4.34 (0.70, 26.98)	0.36 (0.07, 1.82)
Melanoma subtype	*P < .001*	*P < .001*	*P = .141*	*P = .961*	*P = .735*	*P = .665*
SSM	1	1	1	1	1	1
NM	1.40 (0.79, 2.47)	1.17 (0.60, 2.26)	1.51 (0.59, 3.91)	0.93 (0.31, 2.73)	0.77 (0.25, 2.40)	1.20 (0.52, 2.79)
LMM	0.43 (0.28, 0.66)	0.29 (0.18, 0.46)	0.46 (0.24, 0.92)	0.93 (0.44, 1.97)	0.62 (0.28, 1.34)	1.50 (0.85, 2.66)
Other	0.62 (0.31, 1.23)	0.42 (0.20, 0.86)	0.62 (0.21, 1.79)	1.01 (0.30, 3.37)	0.68 (0.20, 2.32)	1.49 (0.60, 3.72)
Unknown	0.66 (0.45, 0.95)	0.60 (0.38, 0.94)	0.87 (0.45, 1.67)	0.75 (0.37, 1.55)	0.69 (0.32, 1.48)	1.09 (0.63, 1.89)
Lesion site	*P < .001*	*P < .001*	*P = .066*	*P = .556*	*P = .646*	*P = .039*
Head and neck	1	1	1	1	1	1
Trunk	3.04 (2.06, 4.49)	2.18 (1.42, 3.37)	2.06 (1.11, 3.82)	1.48 (0.74, 2.95)	1.67 (0.67, 4.06)	1.39 (0.82, 2.37)
Upper limb	2.66 (1.70, 4.2)	2.21 (1.33, 3.65)	2.42 (1.14, 5.14)	1.10 (0.48, 2.54)	1.06 (0.52, 2.17)	1.21 (0.65, 2.25)
Lower limb	1.75 (1.75, 2.67)	3.21 (1.79, 5.75)	1.95 (0.93, 4.07)	0.90 (0.40, 2.01)	0.91 (0.38, 2.17)	0.55 (0.28, 1.07)
Remoteness clinic∗,†	*P = .159*	*P < .001*	*P = .042*	*P = .055*	*P = .013*	*P = .085*
Major cities	1	1	1	1	1	1
Inner regional	1.18 (0.86, 1.62)	1.70 (1.14, 2.44)	2.09 (1.15, 3.82)	0.56 (0.29, 1.09)	0.80 (0.40, 1.59)	0.71 (0.44, 1.12)
Outer regional, remote	1.57 (0.97, 2.55)	3.61 (1.72, 7.55)	0.97 (0.51, 1.85)	1.62 (0.75, 3.5)	3.71 (1.43, 9.63)	0.44 (0.19, 1.02)
SES clinic location‡	*P = .007*	*P = .003*	*P = .203*	*P = .072*	*P = .027*	*P = .194*
Q1 (disadvantaged)	1	1	1	1	1	1
Q2	0.82 (0.42, 1.62)	2.57 (1.20, 5.52)	0.95 (0.32, 2.80)	0.87 (0.25, 2.99)	2.7 (0.75, 9.80)	0.32 (0.12, 0.85)
Q3	0.93 (0.49, 1.76)	1.46 (0.79, 2.69)	0.73 (0.27, 1.94)	1.28 (0.41, 3.97)	2.00 (0.65, 6.14)	0.64 (0.28, 1.48)
Q4	0.47 (0.24, 0.91)	0.75 (0.40, 1.42)	1.99 (0.52, 7.55)	0.24 (0.06, 1.01)	0.38 (0.09, 1.59)	0.63 (0.27, 1.48)
Q5 (most advantaged)	0.56 (0.30, 1.05)	1.08 (0.59, 1.99)	0.60 (0.22, 1.59)	0.94 (0.31, 2.91)	1.82 (0.60, 5.56)	0.52 (0.23, 1.18)
Clinician type	*P < .001*	*P < .001*	*P < .001*	*P = .215*	*P = .001*	*P < .001*
GP	1	1	1	1	1	1
Dermatologist	0.41 (0.30, 0.56)	0.07 (0.04, 0.11)	0.25 (0.15, 0.42)	1.64 (0.93, 2.92)	0.27 (0.14, 0.53)	6.06 (3.56, 10.30)
Surgeon, plastic, other	2.23 (1.31, 3.78)	0.75 (0.36, 1.55)	1.38 (0.59, 3.20)	1.62 (0.61, 4.29)	0.54 (0.18, 1.63)	2.98 (1.23, 7.21)
Practice setting	*P = .009*	*P < .001*	*P < .001*	*P = .086*	*P < .001*	*P < .001*
GP/Skin cancer clinic	1	1	1	1	1	1
Specialist	0.68 (0.51, 0.91)	0.13 (0.08, 0.20)	0.42 (0.25, 0.68)	1.62 (0.94, 2.82)	0.31 (0.16, 0.60)	5.22 (3.12, 8.74)

The following variables were collapsed for univariate and multivariable analysis: (i) Age groups <60, 60-70, and 80+; (ii) Lesion site where anterior trunk and posterior trunk were merged to trunk; and (iii) melanoma subtype in which SSM + other were merged and LMM + unknown were merged. (iv) Remoteness for clinic location where the category remote was merged with outer regional. (iii) Clinician type in which plastic surgeon was merged with surgeon and the category other was removed as it had only 1 value.

*GP*, General practitioner; *LMM*, lentigo maligna melanoma; NM, nodal melanoma; *OR*, odds ratio (95% confidence intervals); *Ref*, referent; *SSM*, superficial spreading melanoma.

∗ Australian Bureau of Statistics. The Australian Statistical Geography Standard (ASGS) remoteness structure. Five categories: major cities, inner regional, outer regional, remote, and very remote.

† All analyses have combined remote and very remote categories due to extremely low numbers in the very remote category.

‡ Australian bureau of statistics. Socio-economic Indexes for Areas (SEIFA) Index of Relative Socio-economic Disadvantage (IRSD). Quintile.

Table IV shows the results from the multivariable regression analyses. Excision biopsies were less likely in patients aged ≥70 (*P* = .016) and those aged ≥80 (*P* = .034). Excision biopsies were less likely in women (*P* < .003). Excision biopsies were more likely in patients with lesions of 0.8-1.0 mm or 2.1-4.0 mm Breslow thickness (*P* = .039). Patients with lentigo maligna melanoma or unknown histopathology were less likely than those with squamous cell carcinoma to undergo excision biopsy than punch biopsy (*P* = .004), and less likely than those with squamous cell carcinoma to undergo excision biopsy than shave biopsy (*P* = .003). Patients with lesions on their upper/lower limbs or trunk were more likely than patients with lesions on the head and neck to receive an excision biopsy than either a punch (*P* < .001) or shave biopsy (*P* < .003).

**Table IV:** Multivariable logistic regression of biopsy type for clinical melanoma (yes/suspicious), *n* = 1812

Characteristic	Excision vs punch *(Ref = punch)*	Excision vs shave *(Ref = shave)*	Excision vs incision *(Ref = incision)*	Incision vs punch *(Ref = punch)*	Incision vs shave *(Ref = shave)*	Shave vs punch *(Ref = punch)*
Age (y)	*P = .616*	*P = .034*	*P = .016*	*P = .164*	*P = .982*	*P = .065*
<60	1	1	1	1	1	1
60-69	0.77 (0.51, 1.16)	0.81 (0.46, 1.44)	0.58 (0.27, 1.23)	1.09 (0.46, 2.57)	0.90 (0.32, 2.55)	0.84 (0.42, 1.69)
70-79	0.81 (0.53, 1.23)	0.47 (0.27, 0.80)	0.34 (0.17, 0.68)	2.04 (0.92, 4.54)	0.85 (0.34, 2.13)	1.64 (0.84, 3.21)
80+	0.86 (0.52, 1.41)	0.57 (0.31, 1.07)	0.38 (0.18, 0.83)	2.04 (0.85, 4.91)	0.99 (0.36, 2.71)	2.09 (0.96, 4.54)
Sex	*P = .003*	*P = .355*	*P = .701*	*P = .175*	*P = .599*	*P = .026*
Males	1	1	1	1	1	1
Females	0.60 (0.43, 0.84)	0.81 (0.52, 1.26)	0.89 (0.50, 1.59)	0.65 (0.35, 1.21)	0.83 (0.40, 1.69)	0.55 (0.33, 0.93)
Breslow thickness	*N/A*	*P = .039*	*N/A*	*N/A*	*P = .122*	*P = .006*
0.1-0.7 mm	--	1	--	--	1	1
0.8-1.0 mm	--	2.37 (1.14, 4.93)	--	--	2.46 (0.85, 7.10)	0.24 (0.10, 0.59)
1.1-2.0 mm	--	1.46 (0.78, 2.74)	--	--	1.94 (0.76, 4.95)	0.61 (0.30, 1.27)
2.1-4.0 mm	--	1.46 (0.78, 2.74)	--	--	1.94 (0.76, 4.95)	0.61 (0.30, 1.27)
>4.0 mm	5.04 (0.87, 29.34)	--	--	4.76 (0.54, 42.12)	0.16 (0.02, 1.14)	5.04 (0.87, 29.34)
Melanoma subtype	*P = .004*	*P = .003*	*P = .689*	*N/A*	*N/A*	*N/A*
SSM	1	1	1	-	-	-
NM	1.32 (0.73, 2.41)	0.51 (0.20, 1.31)	1.64 (0.61, 4.44)	-	-	-
LMM	0.53 (0.32, 0.86)	0.41 (0.23, 0.73)	0.76 (0.36, 1.61)			
Other	0.53 (0.25, 1.09)	0.34 (0.14, 0.83)	0.70 (0.23, 2.15)			
Unknown	0.59 (0.40, 0.88)	0.46 (0.27, 0.78)	0.89 (0.45, 1.78)			
Lesion site	*P < .001*	*P = .003*	*P = .084*	*N/A*	*N/A*	*P = .418*
Head and neck	1	1	1	-	-	1
Trunk	3.57 (2.31, 5.53)	2.49 (1.46,4.25)	2.22 (1.13, 4.37)	-	***-***	1.07 (0.55, 2.07)
Upper limb	3.14 (1.92, 5.13)	2.40 (1.31,4.40)	2.43 (1.08, 5.44)	-	-	1.15 (0.54, 2.43)
Lower limb	2.02 (1.26, 3.25)	2.83 (1.41, 5.65)	1.73 (0.77, 3.90)	-	-	0.60 (0.27, 1.31)
Remoteness clinic∗,†	*P = .467*	*P = .636*	*P = .383*	*P = .866*	*P = .486*	*P = .405*
Major cities	1	1	1	1	1	1
Inner regional	1.02	0.98	1.22	1.02	0.82	0.74
Outer regional, remote	0.54 (0.18,1.56)	1.76 (0.50, 6.19)	0.43 (0.10, 1.96)	2.45 (0.09, 65.35)	4.03 (0.28, 58.31)	0.26 (0.03, 1.98)
SES clinic location‡	*P = .060*	*P = .051*	*P = .230*	*P = .251*	*P = .054*	*P = .027*
Q1 (disadvantaged)	1	1	1	1	1	1
Q2	1.45 (0.64, 3.27)	3.56 (1.39, 9.15)	1.05 (0.29, 3.82)	0.93 (0.20, 4.43)	2.99 (0.58, 15.42)	0.20 (0.06, 0.68)
Q3	1.83 (0.86, 3.89)	2.67 (1.21, 5.88)	0.82 (0.26, 2.57)	1.44 (0.35, 5.94)	2.57 (0.64, 10.27)	0.43 (0.15, 1.23)
Q4	0.90 (0.40, 2.02)	2.03 (0.88, 4.65)	2.61 (0.58, 11.74)	0.37 (0.06, 2.20)	0.66 (0.12, 3.66)	0.37 (0.12, 1.12)
Q5 (most advantaged)	0.97 (0.44, 2.15)	2.91 (1.27, 6.66)	0.71 (0.21, 2.43)	1.31 (0.28, 6.01)	3.93 (0.90, 17.12)	0.21 (0.07, 0.63)
Clinician type	*P < .001*	*P < .001*	*P < .001*	*N/A*	*N/A*	*P < .001*
GP	1	1	1	-	-	1
Dermatologist	0.48 (0.34, 0.69)	0.08 (0.05, 0.14)	0.31 (0.18, 0.55)	*-*	-	6.86 (3.68, 12.79)
Surgeon, plastic, other	3.11 (1.75, 5.54)	0.85 (0.39, 1.87)	1.94 (0.78, 4.82)	-	-	4.38 (1.60, 12.05)
Practice setting	*N/A*	*N/A*	*N/A*	*P = .146*	*P = .004*	*N/A*
GP/skin cancer clinic	-	-	-	1	1	-
Specialist	-	-	-	1.62 (0.85, 3.10)	0.32 (0.15, 0.70)	-

Multivariable model includes all the significant (*P* value ≤.20) variables from the univariate analysis, plus age, sex, SES, and remoteness for patients and doctors. Because of confounding between practice setting and clinician type, in the case of both variables being statistically significant at *P* ≤ .2 in the univariable analysis, the variable with the smallest P value was carried forward to the multivariable model. Blank cells (--) indicate the variable was not retained in the multivariable analysis because it did not meet the cut-off criteria of *P* ≤ .20. Clinically melanoma was not included in the multivariable regression as it was considered a stratification variable. The following variables were collapsed for univariate and multivariable analysis: (i) Age groups <60, 60-70, and 80+, (ii) Remoteness for patient and remoteness for doctor in which the category remote was merged with outer regional, (iii) IRSD for patient and IRSD for doctor in which categories Q1 + Q2 + Q3 were collapsed into 1 group as were Q4 + Q5, (iv) Clinician type in which plastic surgeon was merged with surgeon and the category other was removed as it had only 1 value, (v) lesion site where anterior trunk and posterior trunk were merged to trunk, and (vi) melanoma subtype in which SSM + other were merged and LM + unknown were merged.

*GP*, general practitioner; *LMM*, lentigo maligna melanoma; *NM*, nodal melanoma; *OR*, odds ratio (95% confidence intervals); *Ref*, referent; *SSM*, superficial spreading melanoma.

∗ Australian Bureau of Statistics. The Australian Statistical Geography Standard (ASGS) remoteness structure.Five5 categories: major cities, inner regional, outer regional, remote, and very remote.

† All analyses have combined remote and very remote categories due to extremely low numbers in the very remote category.

‡ Australian bureau of statistics. Socio-economic Indexes for Areas (SEIFA).

### Associations by type of health professional

Compared with GPs, dermatologists were less likely to undertake excision biopsy than punch, shave, or incision biopsy (*P* < .001). Surgeons were more likely than GPs to perform both excision biopsy (*P* < .001) and shave biopsy (*P* < .001) than punch biopsy. Finally, specialist clinics were less likely to perform incision biopsies than shave biopsies (*P* = .004) when compared with GP or skin cancer clinics.

## Discussion

In this cohort, just under 1-quarter (24%) of biopsies for clinically suspicious melanomas were partial biopsies including punch, shave, or incision biopsies that were not recommended in the guidelines. Factors positively associated with receipt of excision biopsies included younger age, male sex, melanomas on the trunk or limbs, intermediate Breslow thickness, and treatment by a surgeon. The type of diagnostic biopsy was not related to patient or clinic remoteness. Similarly, SES of patient residence was not associated with biopsy type, and SES of clinic location did not affect the choice of excision biopsy vs other biopsy types.

As noted previously, no other Australian studies have specifically examined issues related to access to specialist clinicians performing melanoma biopsies; hence, Australian comparators are not available. Evidence of variation in biopsy type in other countries is also scarce. One study in the United States analyzing data from a national database, with 48% of melanoma cases (*n* = 126,000), found an increased likelihood of excision biopsy in rural or urban areas compared with metropolitan areas.^15^ Distance from the facility was also a factor, with an increased likelihood of excision biopsy for those treated in facilities between 201 and 1000 miles (324-1609 km) from the patient’s home, compared with 1 and 200 miles.^15^ However, an important limitation of that study was that patients may occasionally have had an initially unrecorded partial biopsy that led to a follow-up excisional biopsy.^15^

It could be speculated that patients living in more advantaged areas have greater access to specialist dermatologists, who may have the expertise and clinical judgment required to perform nonexcisional biopsies while still delivering diagnostic accuracy for patients who may benefit from less invasive techniques. Our results clearly showed that GPs, when compared with dermatologists, were significantly less likely to undertake excision biopsies than punch, shave, or incision biopsies. It is likely that rural clinics are staffed by GPs who perform excision biopsies on more lesions than recommended because they are not trained in skin cancer and are overly cautious.

GPs play an important role in melanoma management in Australia^16^, ^17^, ^18^; just over half of the patients in this study cohort were treated by GPs working in nonspecialist centers. The extent to which clinicians adhere to melanoma biopsy guidelines is likely to be determined by their experience and expertise in managing skin cancers. A 2018 Australian review of 2013-2014 Medicare data by Wu et al^16^ reported the mean excision rate for GPs to be 20 times lower than that of dermatologists. The absence of data on the numbers of nevi (histopathologically proven) biopsied during our study period made it difficult to understand whether the biopsy type preferences for each group of clinicians (GPs, dermatologists, and surgeons) were generally applied regardless of the degree of clinical concern for melanoma vs nevus.

A subsequent study using a subset of 2013 data from the Skin Cancer Audit Research Database concluded that diagnostic and therapeutic management of 589 melanoma patients by GPs was closely aligned with the then current (2008) guidelines.^17^^,^^18^ However, a major limitation of this latter study was selection bias, whereby the majority of participating GPs had a Master’s degree or PhD in the field of skin cancer and had each excised an average of 23.6 melanomas in the year. This is substantially higher than the national average of 0.7 per year for GPs, 7.5 for surgeons, and 13.8 for dermatologists, as reported by Wu and colleagues.^16^ The importance of clinical experience is reflected in an analysis of data on surgical margins and sentinel lymph node biopsy practices from the same Melanoma Patterns of Care Study data set, which demonstrated better concordance with guideline recommendations among clinicians who treated 30 or more melanomas per year. The authors suggested that minimum caseload thresholds for clinicians operating on melanoma patients may be desirable.^19^

A 2020 Victorian study found that the choice of biopsy technique was not related to the degree of suspicion of melanoma.^20^ Furthermore, there were similar use patterns for excisional biopsies across GPs, dermatologists, and surgeons, regardless of melanoma suspicion.^20^ Similarly, an anonymous survey of 285 GPs in the Australian Capital Territory, involving hypothetical scenarios, found 45% made non–guideline-based choices in terms of referral of suspicious melanocytic lesions or the type of biopsy they used and suggested that GP characteristics (level of training, part-time or full-time employment, specialization in skin cancer management, routine use of dermatoscopy, and mean number of biopsies per month) had a strong impact on the decision to refer and the choice of biopsy technique.^21^ Ng and colleagues reported that after adjusting for clinical and histopathological factors, GPs incurred increased odds of misdiagnosis and adverse outcomes compared with dermatologists.^2^ However, an Australian prospective study using data from the Skin Cancer Audit Research Database demonstrated that GPs who subspecialize in skin cancer have higher use of dermatoscopy and diagnose melanoma with greater accuracy than their generalist GP counterparts.^22^

Submission of an adequately excised biopsy specimen is not the only determinant of diagnostic accuracy. A US editorial noted poorer GP performance may be related to GPs sending biopsies to general pathologists, whereas dermatologists use more specialized dermatopathologists.^23^ Furthermore, several studies have highlighted the difficulty of making accurate melanoma diagnoses when the pertinent clinical information is not provided by whichever clinician performs the biopsy.^20^^,^^24^, ^25^, ^26^, ^27^, ^28^

### Strengths and limitations

No previous Australian studies have specifically examined the variation in the provision of recommended diagnostic biopsy techniques for melanoma. This research has used data from the Melanoma Patterns of Care study, a population-based, observational questionnaire study of GPs’ and specialists’ management of melanoma patients residing in NSW, the most populous state of Australia. This study remains the only population-based Australian cohort to contain information about the type of diagnostic biopsy and factors associated with best-practice biopsy procedures. Of note, a strength in our study was the full visualization of the care pathway from the initial tissue sample generating a melanoma notification to definitive treatment.

Generalizability of these findings may be limited by the length of time since data collection (2006-2007). Diagnostic biopsy patterns may have changed since then, with shave biopsies increasingly used to diagnose melanomas.^10^ In our study, shave biopsies of invasive lesions comprised 8.4% of all biopsy specimens, close to the 2005 figure of 9% from a 2019 study using Victorian Cancer Registry data. In 2015, the Victorian Cancer Registry data (*n* = 1200) showed that 20% of all invasive melanoma biopsies were shave biopsies, driven by increasing usage among dermatologists and GPs.^10^ These more recent data indicate a growing diversion from the Australian melanoma management guidelines over time and are of concern because shave biopsies have been shown to be associated with high rates of base transection (54%) and T-upstaging (12%) and underestimate tumor thickness by a mean 0.25 mm.^10^ Importantly, these shave biopsy traits have serious clinical implications,^29^ including an increased risk of misdiagnosing a melanoma as a benign tumour^2^ and inaccurate staging of diagnosed melanomas.^5^

Generalizability may also be limited by the focus on one state in Australia. Other study limitations include the possibility of reporting errors associated with reported data, as well as a missing questionnaire and pathology data (eg, in some cases in which the melanoma subtype was not stated).

## Conclusions

Our data suggest that adherence to national biopsy guidelines for melanoma was suboptimal in NSW, Australia. However, data were lacking on the number of cases in which cosmetic or healing concerns were likely to have influenced the choice of partial biopsy. To gain a deeper understanding of factors predicting choice of biopsy type and potential unwarranted variation, future research should focus on specific barriers and enablers to enhance guideline adherence including distance traveled to access biopsy; type of clinician seen, their relative biopsy rates, and their level of training; and associated out-of-pocket costs for patients treated in private sectors.

## Conflicts of interest

None disclosed.
